# Chondroradionecrosis of the Larynx in a Patient with Laryngeal: A Case Report

**Published:** 2017-05

**Authors:** Aslan Ahmadi, Mohammad Mahdi Salem, Mahdi Safdarian, Shahriar Ilkhani, Roghaieh Hamidian, Mostafa Cheraghipour, Ali Daneshvar, Farzad Izadi

**Affiliations:** 1 *ENT-Head and Neck Surgery Research Center, Hazrat Rasoul Akram Hospital , Iran University of Medical Science, Tehran, Iran.*

**Keywords:** Actinomycosis, Fistula, Larynx

## Abstract

**Introduction:**

Actinomycosis of the larynx has been rarely reported in the literature and usually occurs in patients with a history of laryngeal carcinoma and radiation therapy. However, its co-existence with chondroradionecrosis due to radiotherapy is even rarer. The most common site of infection is the cervicofacial region, especially in the submandibular area.

**Case Report:**

Here we report a 63-year-old male with a history of chemoradiotherapy because of laryngeal cancer 1 year earlier who presented with laryngeal actinomycosis. After prolonged penicillin-based treatment, the patient underwent thyroid cartilage defect reconstructive surgery because of a laryngocutaneous fistula due to chondroradionecrosis. The diagnosis, work-up, and management of the case are discussed, as well as a review of the literature.

**Conclusion:**

Although actinomycotic infection of the larynx is rare, it should be considered in the differential diagnosis of laryngeal complaints, especially in immunocompromised patients.

## Introduction

Actinomycosis has rarely been reported to involve the larynx, and usually occurs in patients with a history of laryngeal carcinoma and radiation therapy (1). The most common site of infection is the cervicofacial region, especially in the submandibular area (2). It is usually caused by Actinomyces israelii, an anaerobic gram-positive organism that is normally present in the oral cavity. Here we report a case of laryngeal actinomycosis in a patient with a history of chemoradiotherapy because of laryngeal cancer that compromised the laryngocutaneous fistula due to chondroradionecrosis. The diagnosis, treatment, and method of reconstructive surgery are discussed.

## Case Report

A 63-year-old male who first presented with dysphonia with a diagnosis of early-stage glottic cancer (clinical stage: T2) refused to give consent for surgery and instead underwent chemoradiation. The chemoradiation therapy consisted of six chemotherapy and 33 radiotherapy sessions, with a total dose of 70 Gy (at 2.0 Gy/fraction). One year after treatment, the patient complained of neck swelling with mild dysphagia as well as fever and chills, accompanied by a suppurative malodorous discharge from three neighboring fistulas, located at the anterior of the neck.

A thorough work-up was conducted to exclude vascular disorders or recurrence of malignancy. Direct laryngoscopy showed swelling of true and false vocal cords on both sides, and a biopsy was taken. Debridement was performed under anesthesia, and cultures and pathology specimens were sent to the laboratory, in addition to fungus smears from the fistula. Pathological examinations of all samples were negative for recurrence of malignancy. Microbiological specimens were also negative. The patient underwent empiric therapy based on consultations with infectious diseases specialists. Histological examination of biopsies revealed actinomycotic colonies in the biopsy specimen with sulfur granuloma. 

The patient was treated with intravenous penicillin following by long-term oral penicillin therapy for 6 months.

 The anterior cervical infection and malodorous discharge showed a good response to antibiotic treatment and the inflammation gradually resolved with long-term antibiotic therapy.

**Fig 1:**
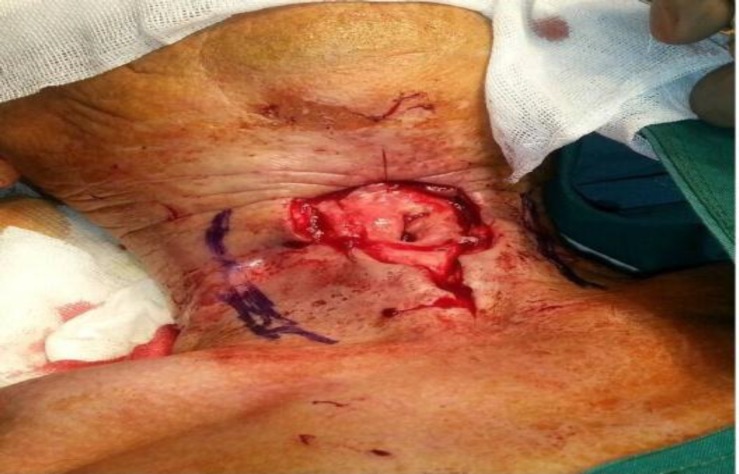
Under general anesthesia, margins of needed skin were removed

However, due to incomplete closure of the fistulas and an 8-mm defect in the thyroid cartilage, laryngoscopy and multiple biopsies of the stoma were performed to rule out malignancy. Radiologic and clinical signs were consistent with a diagnosis of laryngeal chondroradionecrosis. Because of laryngo- cutaneous fistula due to chondroradionecrosis, the patient underwent thyroid cartilage defect reconstructive surgery using nasal septum cartilage graft, and the cutaneous defect was repaired with pectoralis major myocutaneous flap transposition. Under general anesthesia, and after appropriate preparation of the surgery sites, the margins of the skin were removed by a senior surgeon (Fig. 1). 

**Fig 2:**
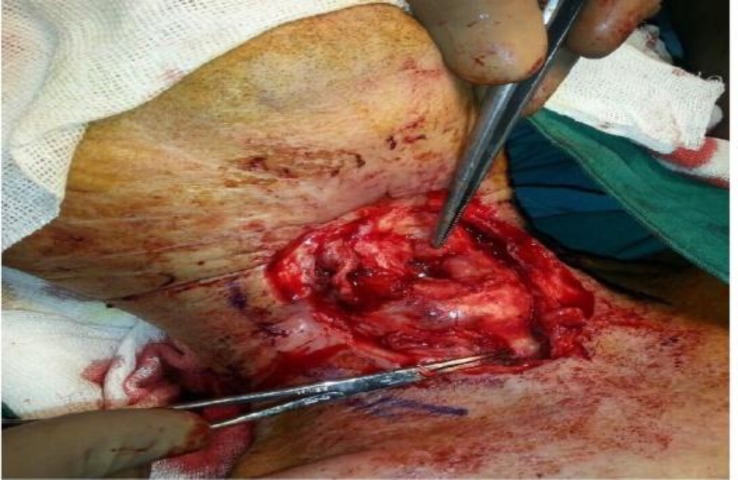
The site of fistula and marginal skin was debrided until bleeding occurred. Fibrotic tissue and necrotic cartilage were also resected

The skin was notably thickened and normal surgical plans were unidentifiable due to previous radiotherapy and long-term infection. The site of the fistula and marginal skin was debrided until bleeding occurred. Fibrotic tissue and necrotic cartilage were also resected (Fig. 2).

**Fig 3:**
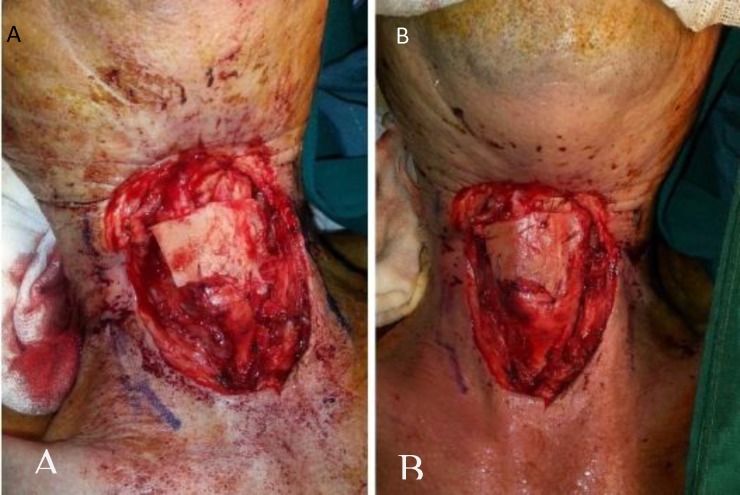
A: Cartilage and its attached bony part from the perpendicular plate of the ethmoid bone was placed on the defect.B: Cartilage was sutured and fixed to the lateral sides

Endolaryngeal mucosa was normal. True vocal cord remnants, surviving after radiation and multiple biopsies, were visible on both sides. Arytenoids were sutured to the lateral sides in order to prevent mucosal prolapse. By measuring the size of the cartilage and skin defect, reconstructions were planned. A cartilage and its attached bony part from perpendicular plate of ethmoid bone, harvested from the nasal septum, were placed on the defect, sutured and fixed to the lateral sides (Fig. 3A,B). 

**Fig 4:**
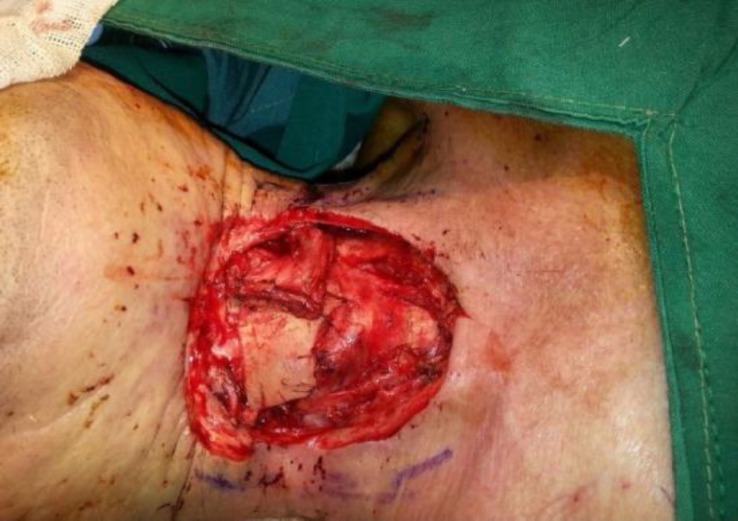
The bony part of graft was covered by rotated sternocleidomastoid muscle flap from the same side

**Fig 5:**
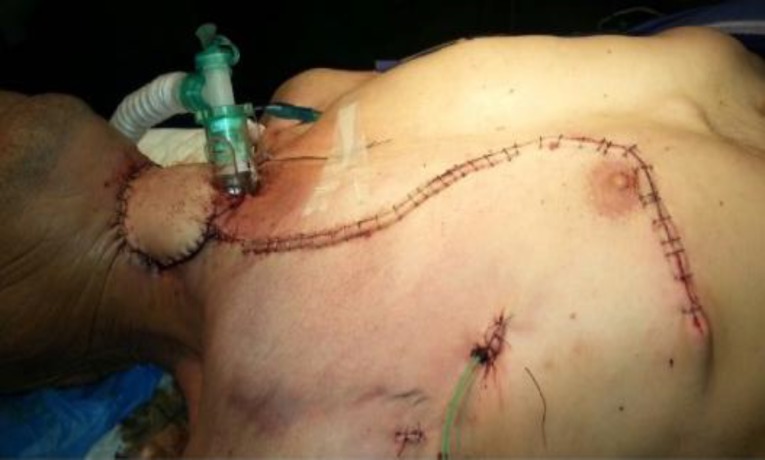
Tracheotomy was performed. An ipsilateral pectoralis major myocutaneous flap was inserted at the defect site. The medial part of the clavicle was resected and vacuum drains were inserted

To assure its survival, the bony part of the graft was covered by the rotated sternocleidomastoid muscle flap from the same side (Fig.4), and a tracheotomy was performed. An ipsilateral pectoralis major myocutaneous flap, of an appropriate size, was harvested and inserted at the defect site. To reduce the tension caused by the previously radiated neck and skin, the medial part of the clavicle was resected. Finally, vacuum drains were inserted (Fig.5). 

The patient stayed in hospital for 6 days and was discharged in a well condition. He was further admitted for monthly follow-up visits. In less than 3 months, the tracheostomy site was closed. Six months after reconstructive surgery, the patient’s airway was patent and video stroboscopy showed normal true vocal cords, false vocal cords, and arytenoid movement, and small granulation tissue on the endolaryngeal mucosa.

## Discussion

Actinomycosis is characterized by a subacute or chronic suppurative and granulomatous process caused by anaerobic or microaerophilic/capnophilic bacteria, mainly within the Actinomyces species (3). Many organs and tissues in the human body can be affected by the actinomycosis. The most common forms of the disease are cervicofacial, thoracic, abdominal, and pelvic actinomycosis; however, infections of the central nervous system, bones and skin, larynx, urinary bladder, and even sepsis have been reported (3). Actinomyces virulence is low, with few virulence factors such as fimbriae, peptidoglycan and biofilm production (4). Most commonly, actinomycosis presents as slowly progressive, but occasionally the infection is acute and rapidly progressive (5). Clinical signs of actinomycosis can be nonspecific, frequently including indolent infiltration with abscesses and draining sinuses, which can mimic other diseases, most often malignancy (1). Many of the associated publications are case reports (5,6); consequently there are difficulties in diagnosis of the infection and in choosing the treatment regimen.

Clinical observation is the start of the diagnostic process. For instance, cervicofacial actinomycosis should be suspected in patients with persistent or recurrent head and neck swelling, especially in the presence of poor dental hygiene and fistulas (sinus tracts) yielding sulfur granules (7). Diagnosis of actinomycosis can be very difficult and is frequently delayed owing to prolonged and nonspecific clinical signs (8). Laboratory findings, showing mild leukocytosis as well as increased C-reactive protein levels and erythrocyte sedimentation rate can be observed (9). Microbiological diagnosis of actinomycosis is the isolation of the causative agent from a sterile body site. The best clinical specimens are deep needle aspirates, sulfur granules from draining sinuses, and tissue biopsy specimens. Conversely, swabs, urine, sputum, or bronchial washing specimens are unsuitable (8,10). The exudates from the fistulous tracts usually contain yellow granules known as "sulfur granules," which is considered as a hallmark for diagnosis (11).

Most patients, as in our case, have a history of laryngeal carcinoma and radiotherapy and present with an ulceration mimicking a laryngeal cancer relapse, with neither abscess nor sinus tract (1). A review of the literature revealed that actinomycosis of the larynx is often associated with an underlying history of squamous cell carcinoma and therapeutic radiation therapy of the larynx. Diabetes, pregnancy, steroid therapy, or cancer are other predisposing factors (1).

The key principle in the therapy of actinomycosis is to use high doses of antibiotics for a prolonged period (2–6-week intravenous therapy + 6–12-month oral therapy) due to induration of infected tissue, a lack of adequate blood supply, and the resulting poor antibiotic penetration into the tissue (8,12). Antibiotic therapy can be combined with surgical treatment for several purposes. For example, to drain abscesses or to relieve obstruction in some cases. A surgical approach is required in cases of widespread necrotic tissue, fistulas, and sinus tracts, in patients nonresponsive to antibiotic therapy, and to exclude the presence of malignant tumors (13).

Actinomycosis of the larynx represents an unusual presentation for a common bacterium comprising the oral and oropharyngeal florae (14,15). Many patients with laryngeal actinomycosis have a history of laryngeal cancer and symptoms that mimic the tumor or tumor relapse (16). There are few cases reported in the literature of laryngeal actinomycosis occurring primarily in the immunocompromised population (2). Chondroradionecrosis of the larynx is also a rare but serious complication of radiation therapy, sometimes requiring total laryngectomy. Chondroradionecrosis can occur many years after completion of radiotherapy, as reported by Balm et al. in two cases of chondroradionecrosis 11 and 18 years after radiotherapy or by Bekiroglu et al. in a case of a patient with oral cancer who developed chondroradionecrosis 20 years later (17,18).The incidence of chondroradionecrosis was reported to be 5% in a retrospective study of 341 patients with laryngeal and hypopharyngeal carcinomas treated by chemoradiotherapy or radiation therapy alone (19). However, in 1998 Naudo et al. reported this rate to be approximately 0.5% in 190 cases (20). This increasing prevalence may be as a result of changes in radiation therapy protocols for patients with head and neck cancer (21).

An important point is to differentiate chondroradionecrosis from tumor recurrence since the symptoms of necrosis can mimic the recurrence of cancer. The computed tomography (CT) appearance of laryngeal chondroradio- necrosis is nonspecific. Despite repeated negative biopsies, it may be necessary to perform total laryngectomy when there is concern about malignant remnants or when the larynx is nonfunctional. Rowley et al. reported seven cases of laryngectomy due to chondroradionecrosis in whom histology revealed residual or recurrent carcinoma in two cases (22). Salvage laryngectomy, which is defined as total laryngectomy when patients either have a nonfunctional larynx due to chondronecrosis or have a biopsy-proven evidence of recurrent or persistent laryngeal disease after radiation therapy, can be considered in cases of failure of conventional treatments (steroids and antibiotics). Local debridement and hyperbaric oxygen are also reported to be effective in improving the laryngeal function in cases of chondroradionecrosis (21). Halkud reported three cases of chondroradionecrosis treated with salvage laryngectomy and using a pectoralis major myocutaneous flap to cover the fistulous skin defect, as well as one case of treatment with medical and hyperbaric oxygen (23).

Reconstructive surgery with pectoralis major muscle transposition after excision of the necrotic skin of the neck and destroyed thyroid cartilage has been reported to have acceptable outcomes in terms of laryngeal function and cosmetic appearance (17, 18). To our knowledge, this is the first case of self-infection with laryngeal Actinomyces in a patient with a history of chemoradiation therapy who was then compromised with laryngocutaneous fistula due to chondroradionecrosis of the larynx that was repaired using nasal septum cartilage graft and myocutaneous flaps transposition.

## Conclusion

Although actinomycotic infection of the larynx is rare, it should be considered in the differential diagnosis of laryngeal complaints, especially in immunocompromised patients. Management of chondroradionecrosis as a complication of chemoradiation therapy in a patient with laryngeal carcinoma can be challenging for ear, nose, and throat (ENT) and head and neck surgeons. Thyroid cartilage defect reconstructive surgery using a nasal septum cartilage graft and myocutaneous flap transposition may be considered acceptable surgical management.
